# Selection and Characterization of Single-Stranded DNA Aptamers Binding Human B-Cell Surface Protein CD20 by Cell-SELEX

**DOI:** 10.3390/molecules23040715

**Published:** 2018-03-21

**Authors:** Mansoureh Haghighi, Hossein Khanahmad, Abbasali Palizban

**Affiliations:** 1 Department of Clinical Biochemistry, Faculty of Pharmacy and Pharmaceutical Sciences, Isfahan University of Medical Sciences, Isfahan 81746-73461, Iran; ma_haghighi@yahoo.com; 2 Department of Molecular Biology and Genetics, Faculty of Medicine, Isfahan University of Medical Sciences, Isfahan 81746-73461, Iran; h_khanahmad@med.mui.ac.ir

**Keywords:** ssDNA aptamer, CD20, Cell-SELEX, HEK293T cells

## Abstract

The B-lymphocyte antigen (CD20) is a suitable target for single-stranded (ss) nucleic acid oligomer (aptamers). The aim of study was selection and characterization of a ssDNA aptamer against CD20 using Cell-Systematic Evolution of Ligands by Exponential Enrichment (Cell-SELEX). The cDNA clone of CD20 (pcDNA-CD20) was transfected to human embryonic kidney (HEK293T) cells. Ten rounds of Cell-SELEX was performed on recombinant HEK-CD20 cells. The final eluted ssDNA pool was amplified and ligated in T/A vector for cloning. The plasmids of positive clones were extracted, sequenced and the secondary structures of the aptamers predicted using DNAMAN^®^ software. The sequencing results revealed 10 different types; three of them had the highest thermodynamic stability, named AP-1, AP-2 and AP-3. The AP-1 aptamer was the most thermodynamically stable one (ΔGAP-1 = −10.87 kcal/mol) with the highest binding affinity to CD20 (96.91 ± 4.5 nM). Since, the CD20 is a suitable target for recognition of B-Cell. The selected aptamers could be comparable to antibodies with many advantages. The AP-1, AP-2 and AP-3 could be candidate instead of antibodies for diagnostic and therapeutic applications in immune deficiency, autoimmune diseases, leukemia and lymphoma.

## 1. Introduction

Aptamers are biomolecular ligands composed of single-stranded (ss) nucleic acid (DNA or RNA) molecules that are specifically isolated from a random sequence pool via in vitro selection process known as Systematic Evolution of Ligands by Exponential Enrichment (SELEX) [1,2]. These specific molecules are able to target or bind a wide range of specific ligands, metal ions, small molecules, proteins, cell surface antigens and whole cells or tissues [3,4].

On the one hand, aptamers have multiple advantages over antibodies such as smaller size; easy, quick, and reproducible synthesis, with low batch-to-batch variability during mass production; low immunogenicity in the body; low toxicity; and modification flexibility. On the other hand, it is indeed possible to select an aptamer molecule with a unique conformation and high binding affinity, with high specificity to a target molecule [4,5]. Because of these features, the aptamers can be considered as an alternative of antibodies [6]. Further, these characteristics render them ideal probes for the clinical research, diagnostics and for therapeutic applications [6,7].

The B-lymphocyte antigen (CD20) protein is a non-glycosylated phosphoprotein encoded by a member of the membrane-spanning 4A gene family (MS4A) and exposed on the surface of the B-cells [8,9]. This tetra-span membrane antigen-like protein is expressed on precursor and mature B lymphocytes, from the early pre-B to the late B stage, except in normal plasma cells and pro-B cells [10]. The membrane-attached CD20 protein acts as a membrane ion channel specifically, a Ca^2+^ channel that regulates Ca^2+^ concentration following B-cell antigen receptor (BCR) induction, and authorizes the activation of B-cells [11,12]. The protein is involved in B-cell growth, proliferation, and cell cycle signaling by triggering intracellular tyrosine kinase signaling pathways. These functions depend on the various expression of CD20, e.g., the expression of CD20 on B-cell precursor acute lymphoblastic leukemia (BCP-ALL) is 30–50%; and in mature B-ALL or Burkitt-type leukemia/lymphoma, it is 80–90%. This phenomenon is used to facilitate diagnosis and prognosis of different types of B-cell acute lymphoblastic leukemia (B-ALL) [13,14]. For instance, anti-CD20 monoclonal antibody enables the immunophenotyping of B-cell lineages [14].

Along with gaining more knowledge about the role of CD20, antibodies against CD20 have been recently developed to treat various B-cell malignancies [15]. Rituximab is an Food and Drug Administration (FDA) approved anti-CD20 chimeric monoclonal antibody and has significantly improved the outcomes of the Burkitt-type ALL, chronic lymphocytic leukemia (CLL), and Non-Hodgkin’s lymphoma treatments [16]. Monoclonal antibody therapy might constitute an optional approach for the treatment of disorders associated with autoantibody production and immune-mediated illnesses, such as rheumatoid arthritis, systemic lupus erythematosus, pemphigus foliaceus, dermatomyositis, and idiopathic thrombocytopenic purpura, as well as post-transplant lymphoproliferative disorders (PTLD) [14,17]. Furthermore, monoclonal antibody therapy directed against the CD20 antigen does not target plasma cells, and consequently, serum immunoglobulins producing and immunological memory cells [14,18]. There is no competition between plasma and lymphoma cell antigens for binding to rituximab because CD20 antigen is not released from the surface of B-cells and, hence, free CD20 antigen is generally not present in the serum in the soluble form [19]. Therefore, based on these exclusive roles, CD20 antigen is an important target for the diagnosis and treatment of B cell related disease such as autoimmune diseases and B-ALL [2,20].

The sequence of CD20 protein revealed that the extracellular domain of CD20 contains loops located near the cell surface. This would enable targeting of the CD20 membrane protein by aptamers, which are comparable with monoclonal antibodies [11,12]. Though, monoclonal antibodies act differently against each of the CD20 epitopes and there is also a great interest in the production of novel antibodies against CD20, but due to some of the disadvantages of classical antibodies such as thermal instability and immunogenicity, this makes limitation of using antibodies and leading to create alternative molecules resemble to them. Among these alternative molecules, the production of aptamers were considered [21]. Aptamers are desirable because of rapid detection methods, cheap, fast and reliable with no complexity. On the other hand, the cell-selected aptamers against CD20 can provide distinguishable tools in diagnostics and therapeutics. Therefore the design, selection and production of them were desired. In the current study, ssDNA aptamers directed against HEK293T cells expressing CD20 were designed and selected via Cell-SELEX and finally their potency were characterized.

## 2. Results

### 2.1. Over-Expression of CD20 in HEK293T Cells

**Figure 1:**
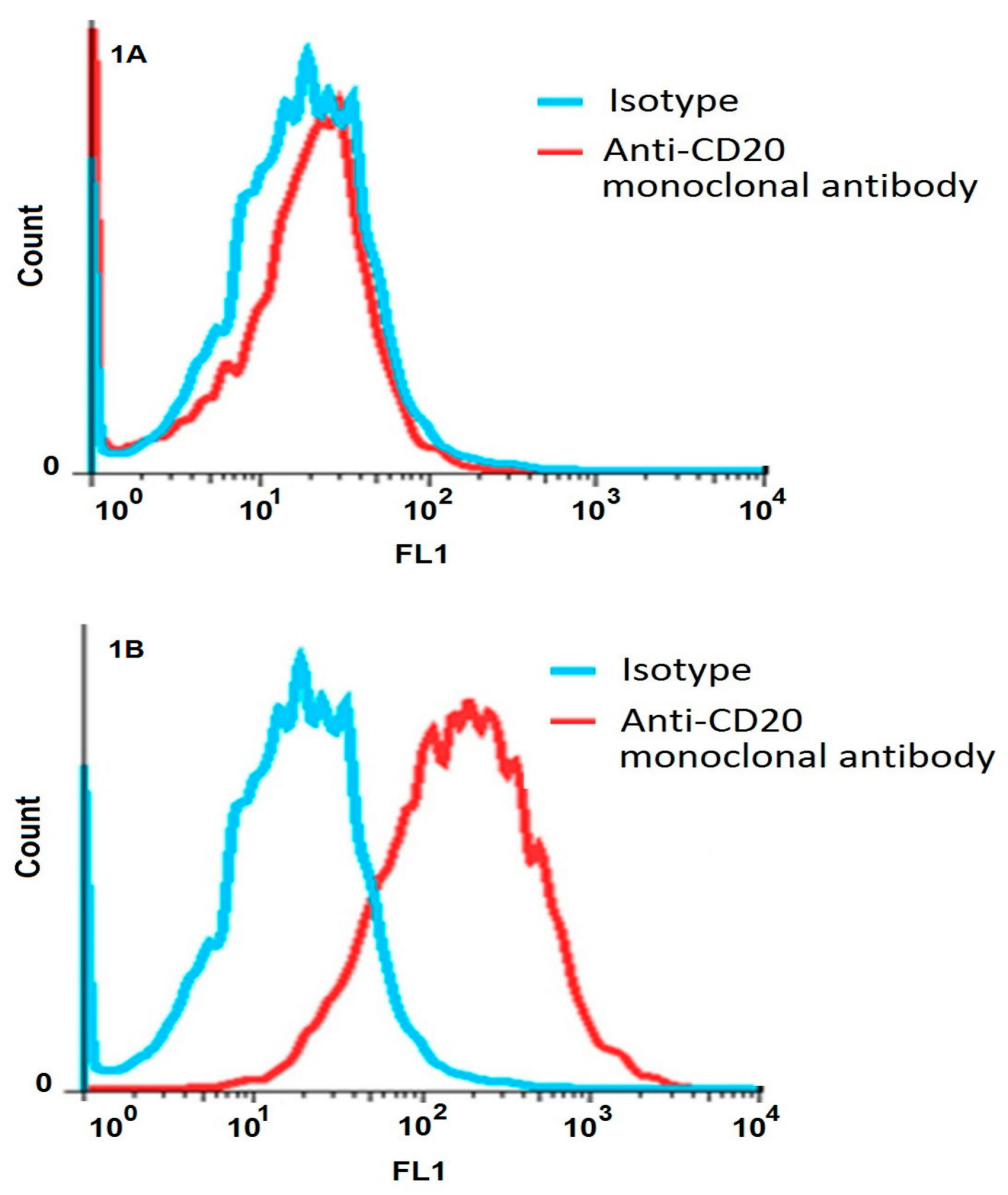
Flow cytometry analysis verified the expression of the CD20 protein on the surface of CD20 + HEK293T cells using Fluorescein Isothiocyanate (FITC)-conjugated anti-CD20 monoclonal antibodies. (**1A**) lack of expression of CD20 on the surface of HEK293T cell as a negative selection, no difference in fluorescent intensity between isotope and anti CD20 antibody; (**1B**) Over-expression of CD20 on transfected CD20 + HEK293T cells was shown. Fluorescent light channel 1 (FL-1).

The CD20-encoding pcDNA3.1/Hygro(+) plasmid (pcDNA-CD20) was used to stably transfect the Human embryonic kidney cells 293 (HEK293T) using Lipofectamine 2000. The generated CD20 + HEK293T cells were used in positive selection rounds of Cell-SELEX, and the untransfected HEK293T cells used as negative selection. Flow cytometry analysis of untransfected HEK293T cells and CD20 + HEK293T cells stained with the isotype and anti-CD20 antibodies confirmed the overexpression of CD20 in the transfected cells, with a maximum yield of 80%. No evidence of membrane CD20 expression in the wild-type HEK293T cells was apparent (Figure 1A,B).

### 2.2. Selection of CD20-Specific Aptamers by Cell-SELEX and Their Characterization

Nowadays various SELEX methods have been developed. In the current study, Cell-SELEX was employed because of its particular advantages. In Cell-SELEX, the targeted proteins are displayed in their native form and aptamers act as high-affinity ligands that bind the target molecules that have post-translational modifications and natural folded conformation on the cell surfaces [22]. In the current study, 11 rounds of Cell-SELEX selection were performed to obtain specific aptamers exhibiting high affinity to the CD20 protein.

From the second round of Cell-SELEX, one negative (counter) selection was performed to remove non-specific oligonucleotides. In addition, after fourth round of Cell-SELEX, the binding affinity of the eluted ssDNA pool to CD20 + HEK293T cells was also evaluated by flow cytometry.

**Figure 2:**
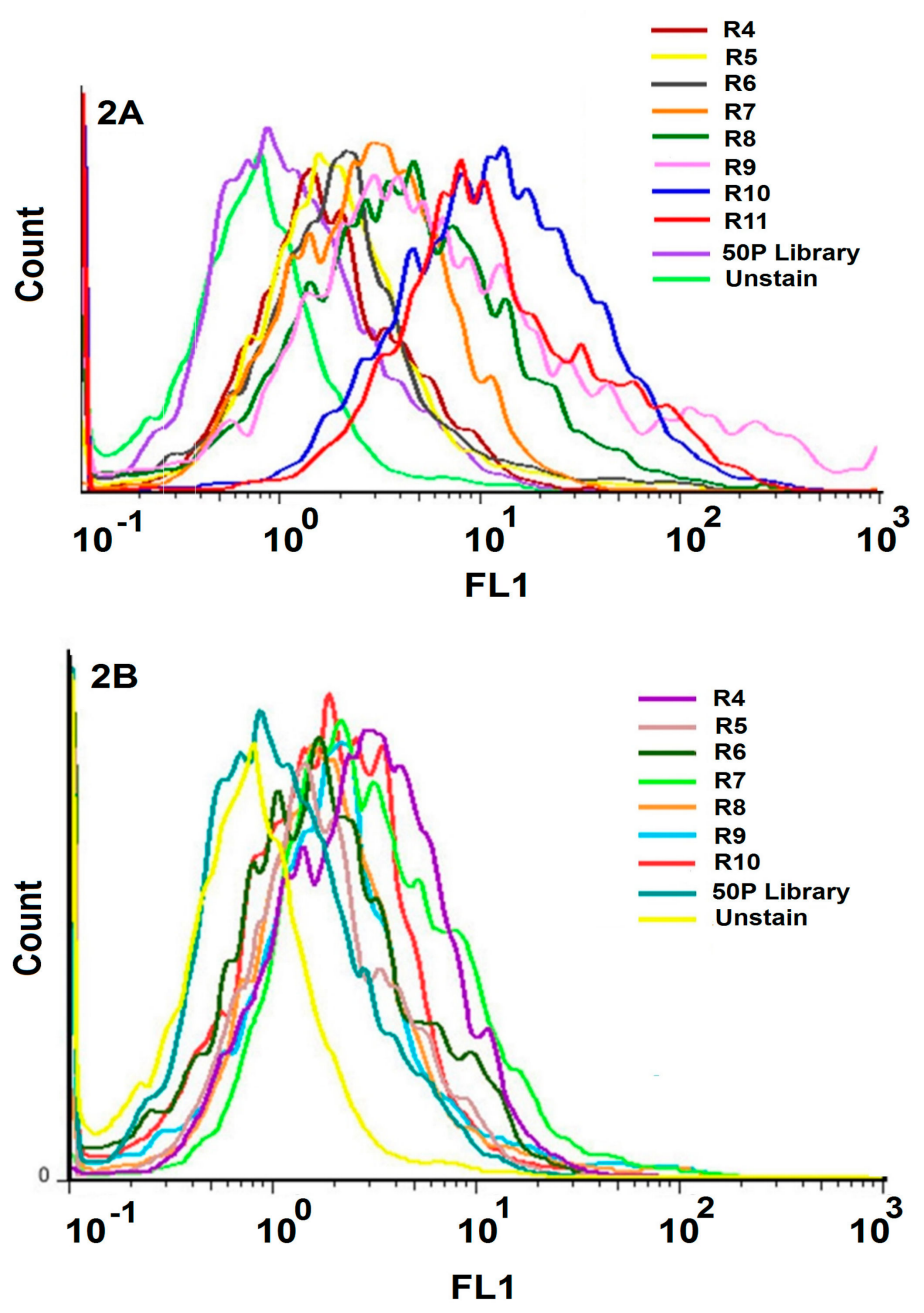
Evaluation of the binding affinity of aptamer pools from each round of Cell-SELEX(R) to the CD20 protein expressed on the surface of HEK293T cells. (**2A**) the binding affinity of ssDNA after seven rounds of Cell-SELEX (rounds 4 to 10) after 11 rounds of Cell-SELEX on CD20 + HEK293Tcells; (**2B**) The binding affinity of pools from seven rounds of Cell-SELEX (rounds 4 to 10) after 10 rounds of Cell-SELEX on HEK293T cells. 50 pmol of the library was used as a negative control. Since the 10th round pool was selected as a final product; the 11th round for HEK293T cells was not applicable to show inside the Figure 2B.

As shown in Figure 2A, gradually increasing fluorescence intensity against the transfected HEK293T was observed with an increasing number of Cell-SELEX rounds. The peaks shifted far away the library peak. This was apparent for the 10th round pool of Cell-SELEX. No detectable shifts were seen with the untransfected HEK293T cells (the negative control, Figure 2B).

Since the peak in the 11th round for CD20 + HEK293T cells moved to the left and subsequently the movement to the right was stopped; so it was preferred to use the 10th round pool as a final product. Therefore, the 11th round for HEK293T cells was not shown in the Figure 2B. These observations clearly demonstrated that specific aptamers with high-binding affinity against CD20 were enriched during Cell-SELEX rounds.

To identify the specifically enriched aptamers, 60 selected clones were analyzed. The individual clones that represented the selected ssDNA pool were amplified and sequenced. The results of sequencing revealed 10 different aptamers that harbored some common conserved regions.

### 2.3. Sequences of CD20-Specific Aptamers and Determination of K_D_ Values

The secondary structure of the selected ssDNA aptamers was next predicted using by the DNAMAN software. The three aptamers with the highest structural thermodynamic stability were AP-1 (ΔGAP-1 = −10.87 kcal/mol), AP-2 (ΔGAP-2 = −10.53 kcal/mol) and AP-3 (ΔGAP-3 = −5.59 kcal/mol). For these three cases showing the highest thermodynamic stability, K_D_ was calculated.

**Figure 3:**
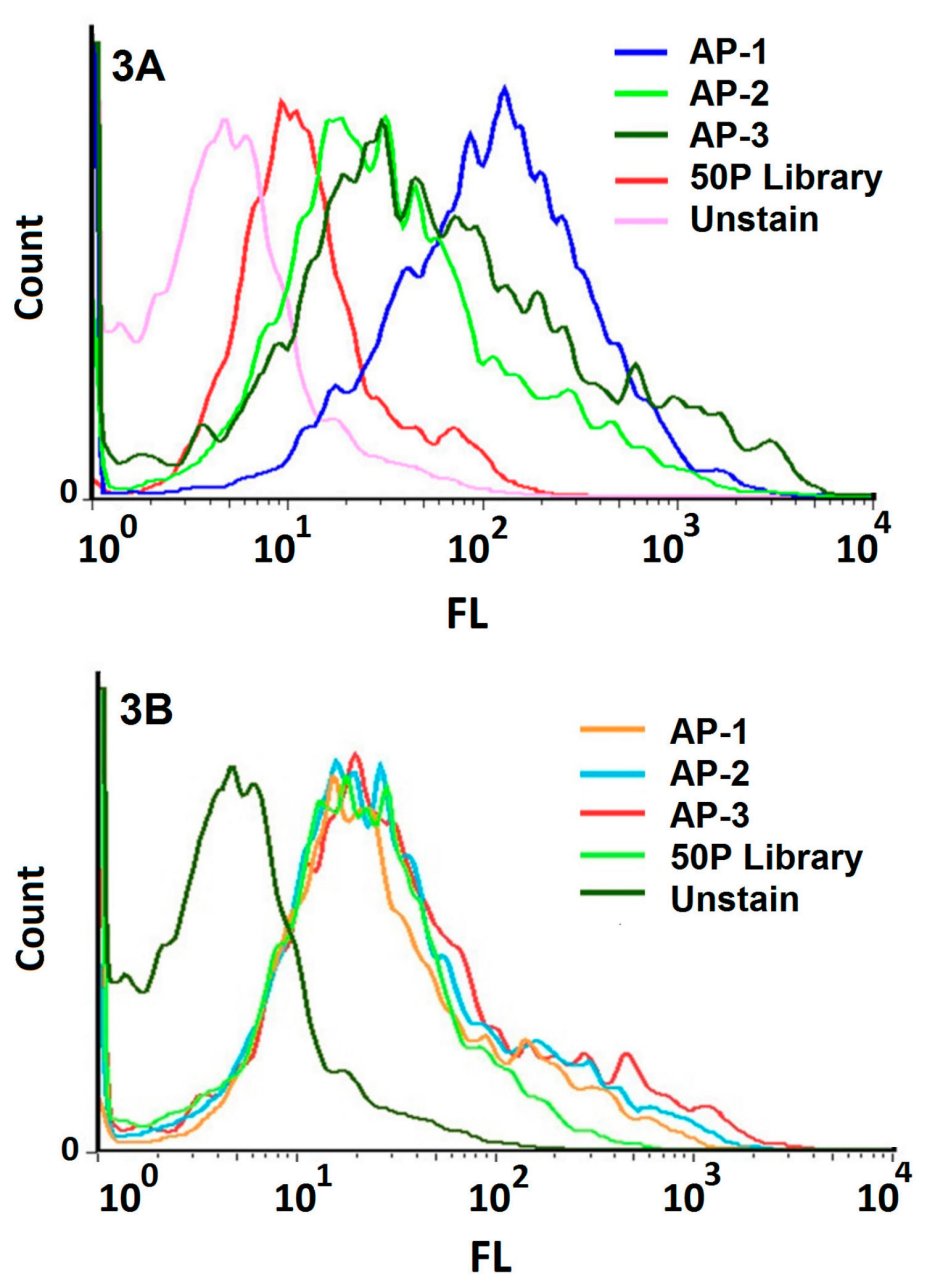
The evaluation of binding affinity of three selected aptamers of pool 10. (**3A**) the binding affinity of AP-1, AP-2 and AP-3 aptamers to CD20 + HEK293T cells and (**3B**) of all three aptamers incubated with the HEK293T cells under the same conditions.

The binding affinities of AP-1, AP-2, and AP-3 to CD20 + HEK293T cells were compared (Figure 3). The fluorescent intensities of the three selected aptamers exhibited a shift to the right compared with the aptamer library. The shift was most pronounced in the case of the AP-1 aptamer. Incubation of the three aptamers with the HEK293T cells under the same conditions did not affect the fluorescent profiles (Figure 3B). We concluded that AP-1 bound the CD20 + HEK293T cells with the maximum binding affinity. As shown in Figure 3, AP-1 bound well to HEK293T cells expressing CD20 (Figure 3A) while it was unable to recognize the untransformed HEK293T cells (Figure 3B).

**Figure 4:**
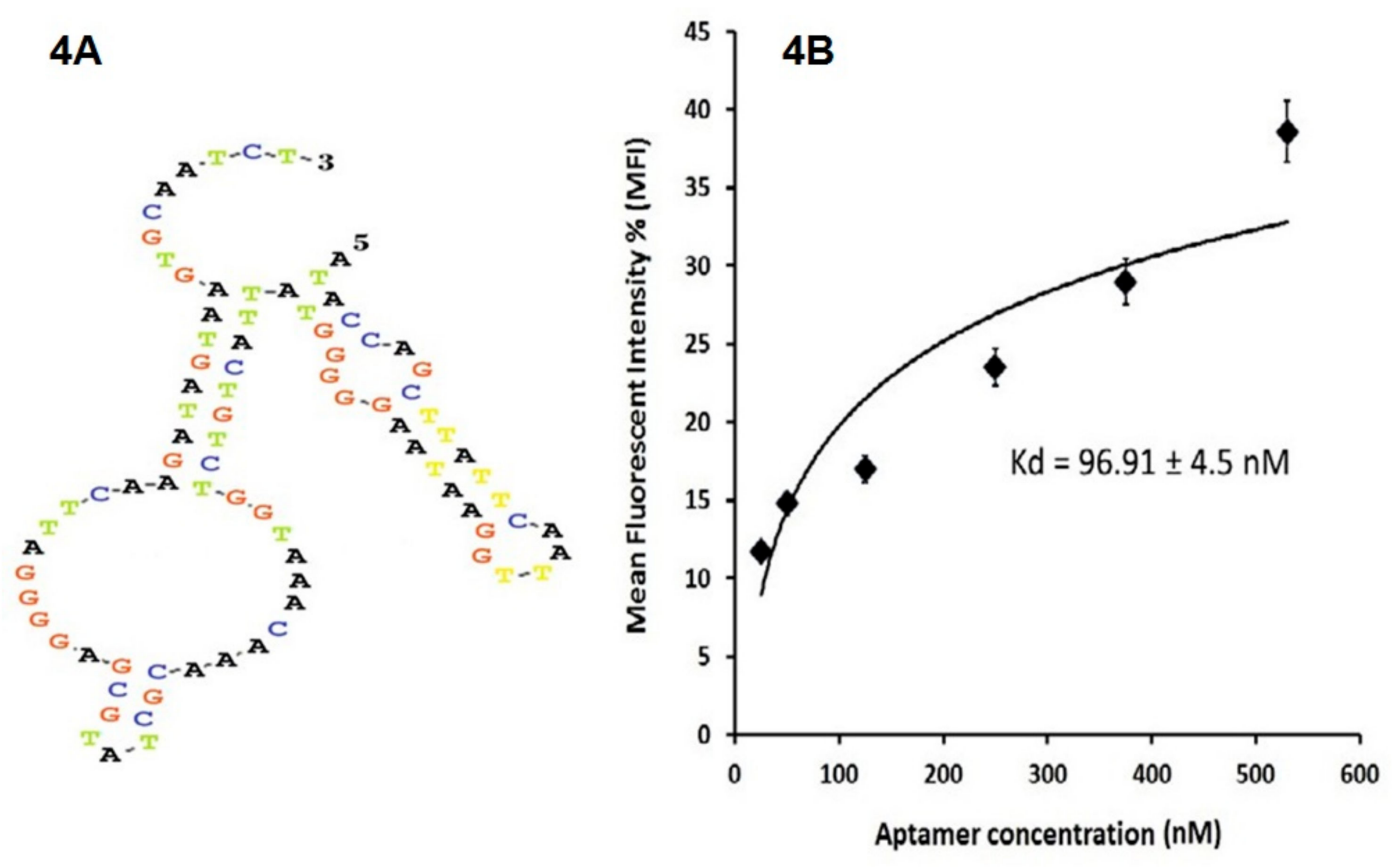

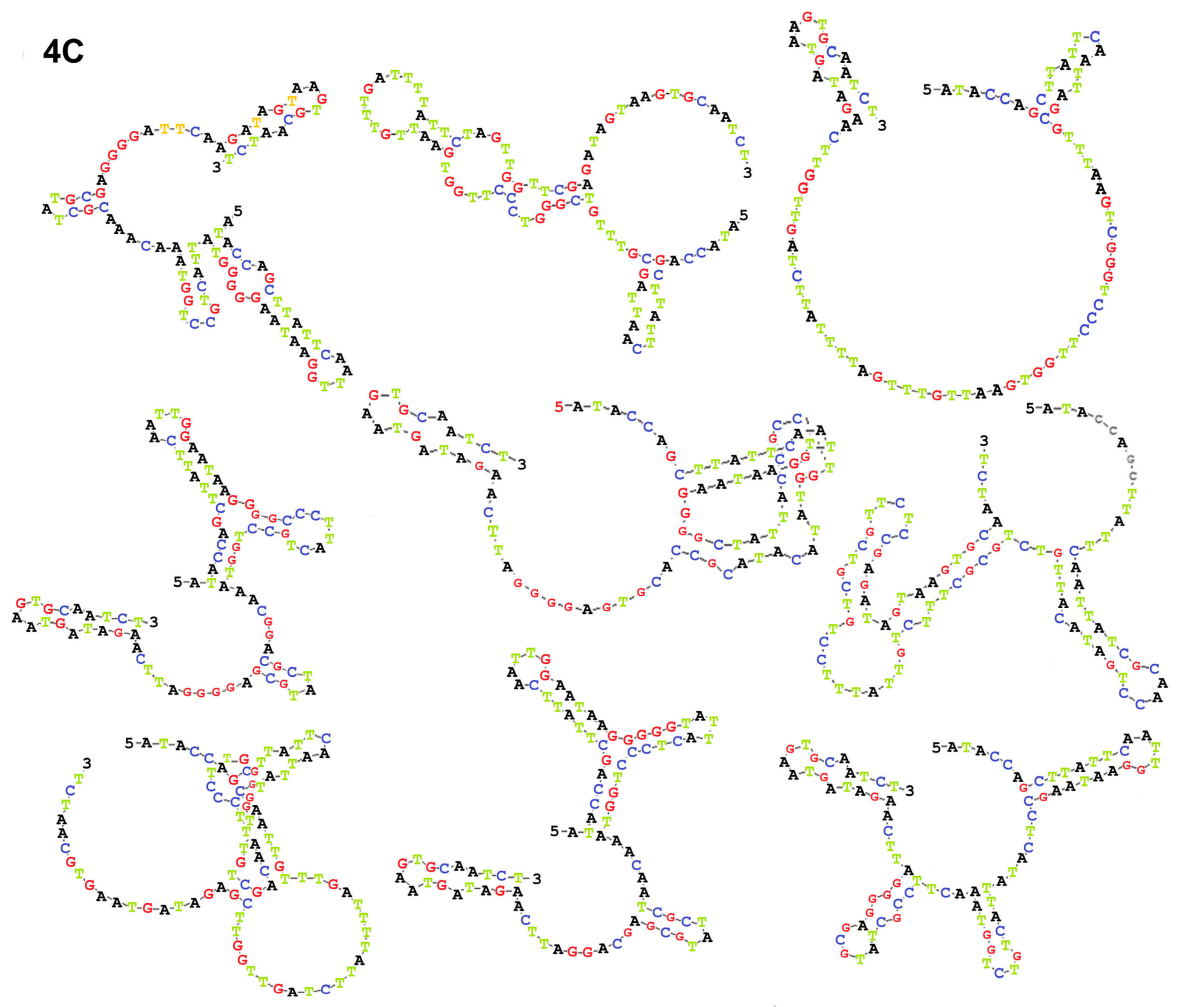
The secondary structure of AP-1 and the curve related to the calculation of K_D_. (**4A**) the secondary structure of the AP-1 aptamer, as predicted by the DNAMAN^®^ software; (**4B**) the correlation between aptamer concentration and MFI used to estimate K_D_; (**4C**) the secondary structure of the nine other aptamers are shown as the conformation predicted by the DNAMAN^®^ software.

The secondary structure of the AP-1 aptamer is shown in Figure 4A. The sequence is: 5′-ATACCAGCTTATTCAATTGGAATAAGGGGGTATTACTGTCTGGTAAACAAACGCTATGCGAGGGGATTCAAGATAGTAAGTGCAATCT-3′. The hairpin usually plays a crucial role in CD20 binding; the calculated minimum free energy of this structure was −10.89 kcal/mol. The AP-1 had the highest binding affinity to the CD20 + HEK293T cells with a K_D_ value of 96.91 ± 4.5 nM (Figure 4B) while the K_D_ for AP-2 and AP-3 is equal to 119.4 ± 8.1 nM and 129.9± 4.0 nM, respectively.

The equilibrium dissociation constant (K_D_) of the aptamer-cell interaction was calculated using the following equation:

Y = B_max_ × X/(K_D_ + X) (1)

where X was the aptamer concentration; Y was mean fluorescence intensity (MFI) X; and B_max_ was maximum MFI.

### 2.4. Evaluation of Selected Aptamer with Clinical Sample

**Figure 5:**
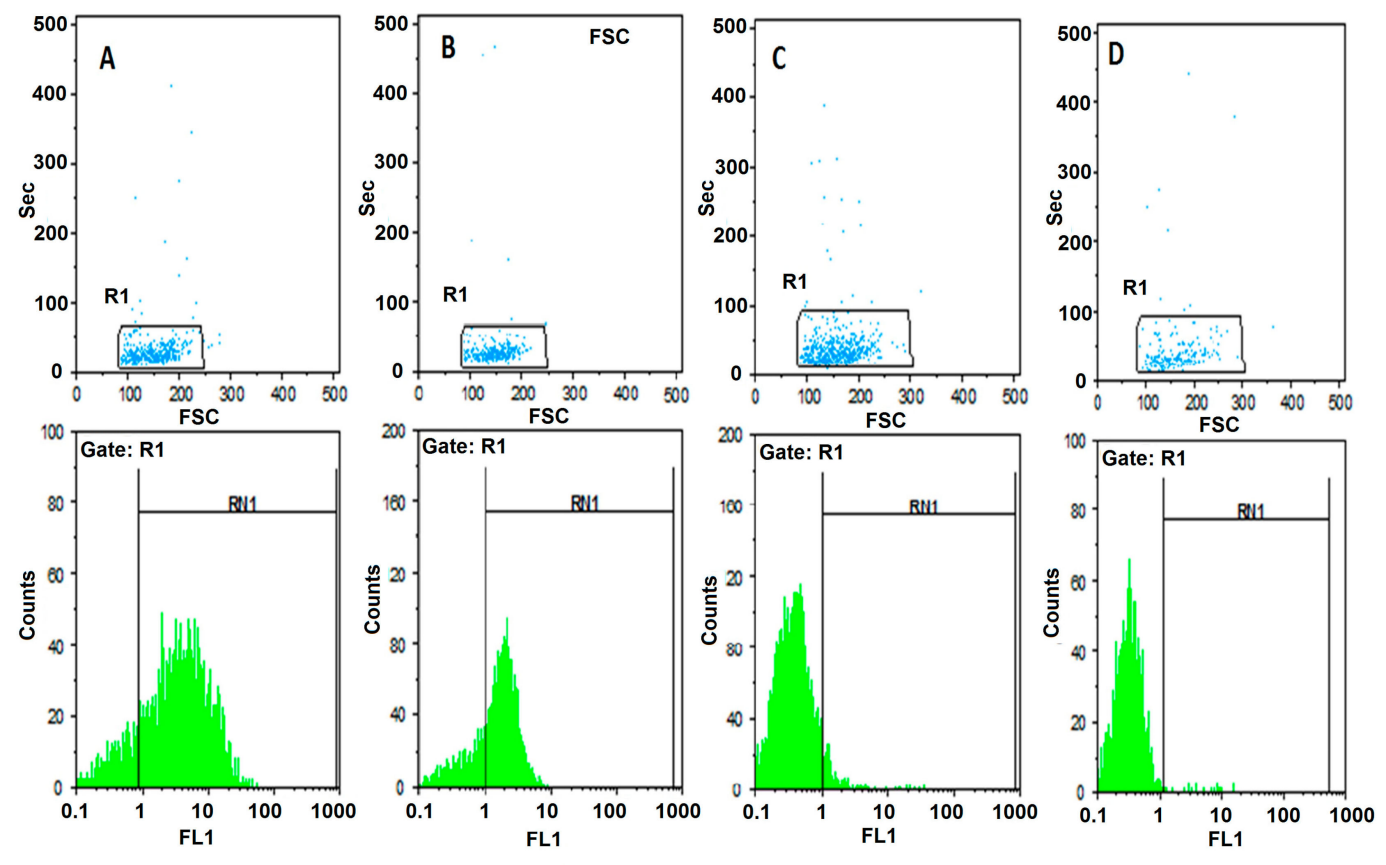
The flow cytometry evaluation of AP-1 aptamer with a negative sample (no expressed CD20) and positive sample (expressed CD20) to compare aptamer and monoclonal antibody. (**A**) the flow cytometric analysis of CD20 expression by Anti-CD20 Antibody incubated with immature bone marrow cells of patient with Pre-B ALL; (**B**) the flow cytometric analysis of CD20 expression by AP-1 aptamer with immature bone marrow cells of patient with Pre-B ALL; (**C**) the flow cytometric analysis of CD20 expression by Anti-CD20 Antibody incubated with immature bone marrow cells of patient with T-ALL; (**D**) the flow cytometric analysis of CD20 expression by AP-1 aptamer incubated with immature bone marrow cells of patient with T-ALL.

In order to characterize the specificity of AP-1 aptamer binding to CD20 with the aims of evaluating the validity of the proposed SELEX strategy; the immunofluorescence experiment was performed with the fluorescent labeled aptamer and compared with anti-CD20 antibody. Two samples of bone marrow from patients with ALL were taken as positive and negative controls. The first sample was selected with Pre-B ALL as a positive control who had a high expression of CD20 revealed by flow cytometric analysis with anti-CD20 monoclonal antibody labeled with Fluorescein Isothiocyanate (FITC) (84.4%) (Figure 5A). It was compared with AP-1 aptamer that was amplified with FITC conjugated primer. In this case a high expression of CD20 was also observed (71.4%) (Figure 5B). The second sample was selected with T-ALL as a negative control who had very low expression of CD20 revealed by flow cytometric analysis with anti-CD20 monoclonal antibody labeled with FITC (6.8%) (Figure 5C). It was compared with AP-1 aptamer that was amplified with FITC conjugated primer. In this case a very low expression of CD20 was also observed (1.5%) (Figure 5D). The comparison of results is shown in Table 1.

## 3. Discussion

The B-lymphocyte antigen CD20 is a 33 to 37-KDa, nonglycosylated phosphoprotein expressed on the surface of mature naive B cells departs from bone marrow to enter the blood. CD20 originally identified as a B-cell surface marker involved in Ca^2+^ channeling, B-cell activation and proliferation [23]. CD20 is not only a marker for recognition of B-cell but also it is a suitable target for treatment [16]. Nucleic acid aptamers are single-stranded RNA or DNA oligonucleotides, from 20 to 90 nucleotides. Aptamers similar to antibodies are able to bind target molecules with high affinity and specificity [1]. Nucleic acid aptamers, termed chemical antibodies, are functionally comparable to antibodies with advantages including; their relatively small molecule in size, ease in production, chemically flexible for structural modification, stability and the most important point lack of immunogenicity. Therefore, aptamers could be replaced instead of antibodies for diagnostic and therapeutic applications in immune deficiency, autoimmune diseases and cancer [24].

SELEX provides us a good and very effective method for producing aptamers.But given that this method does not ignore the complexity of cellular settings; they may not conform to in vivo analyses. In Cell-SELEX, aptamers are chosen in a more natural physiological environment and better matched in in vitro and in vivo analyses. Of course, there are some restrictions in choosing aptamers with this method in Cell-SELEX. An aptamer can be selected against other unwanted targets on a cell surface. To overcome this problem, the same cell that is negative for desired antigen is used for counter SELEX. In current study transfected CD20 + HEK293T and HEK293T were used for SELEX and counter SELEX, respectively [25,26].

The evaluation of transfection of HEK293 cells using flow cytometry by anti-CD20 monoclonal antibodies verified the expression of CD20 only on transfected CD20 + HEK293 cells and not the HEK293 control cells. The ten rounds of alternating Cell-SELEX were made using positive and negative cell lines; we increased stringency during the selection process which led to a regular increase in affinity for positive cells. The results confirmed that pool of 10th round was the highest affinity pool with obviously greater binding to the transfected CD20 + HEK293 cells than the initial library and with the same affinity to the negative cells compared to the initial library. Also aptamers of pool 10th did not show any increase in binding affinity when compared with the untransfected control.

Through Cell-SELEX we use exonuclease III lambda for conversion double stranded DNA to single stranded DNA. There are several methods for conversion of double strand DNA to ssDNA including: (1) Using exonucleas III lambda phage; (2) Separation of leading strand by streptavidin beads and biotinylated revers primer; (3) Imbalance Polymerase chain reaction (PCR) with forward primer [27].

Aptamer pools are certainly heterogeneous; so cloning and sequencing were done with the goal of transforming the pool into single distinct sequences to evaluate their biological considerations including three-dimensional structure, thermodynamic stability and determine their affinity by calculating K_D_. K_D_ represents the affinity between two molecules. In other words, it is the concentration of aptamers that bind half of the target sites on positive cells. For K_D_ calculation we chose aptamers of pool 10 which, according to their spatial structure, exhibited the highest thermodynamic stability; because one of the requirements for an effective binding is the stability of two molecules that are attached.

In order to characterize the specificity of AP-1 aptamer binding to CD20 with the aims of evaluating the validity of the proposed SELEX strategy; the immunofluorescence experiment was performed with the fluorescent labeled aptamer and compared with anti-CD20 antibody. To find that, two samples of bone marrow from patients with ALL was taken as positive and negative controls. The first sample was Pre-B ALL as a positive control who had a high expression of CD20 revealed by flow cytometric analysis with anti-CD20 monoclonal antibody labeled with FITC (84.4%). It was compared with AP-1 aptamer that was amplified with FITC conjugated primer. In this case a high expression of CD20 was also observed (71.4%).

The second sample was T-ALL as a negative control who had very low expression of CD20 revealed by flow cytometric analysis with anti-CD20 monoclonal antibody labeled with FITC (6.8%). It was compared with AP-1 aptamer that was amplified with FITC conjugated primer. In this case a very low expression of CD20 was also observed (1.5%).

It should be noted that the two cases studied were both at the beginning of the diagnostic line at the time of the examination. Over 90% of the studied population gated in flow cytometry were homogeneous and all of them were immature cells.

However, there are lacks of study consist of several crucial factors which delayed the clinical translation of therapeutic aptamers, such as their inherent physicochemical characteristics and lack of safety data left for challenging to approve for therapeutics application.

## 4. Materials and Methods 

### 4.1. In Vitro CD20 Transfection

Stable transfection was performed to express the CD20 antigen (NCBI Accession Number BAE47068) on the surface of human embryonic kidney cells (HEK293T). The CD20 cDNA (909 bases) was synthesized and cloned into the pcDNA-3.1/Hygro(+) vector (Thermo Fisher Scientific, Waltham, MA, USA) between NheI and XhoI sites by Gene Cust Europe (Ellange, Germany). This vector contains the hygromycin B phosphotransferase gene as a selectable marker. Cells were transfected with a linearized plasmid using Lipofectamine 2000 (Life Technologies, Waltham, MA, USA), following the manufacturer’s instructions. The transfected cells were incubated at 37 °C and under atmosphere of 5% CO_2_ in the RPMI-1640 medium (Sigma-Aldrich Company Ltd., Gillingham, UK) containing 10% fetal bovine serum, 100 U/mL penicillin, and 100 µg/mL streptomycin (Thermo Fisher Scientific). After 48-h incubation, the culture medium was refreshed and the cells were treated with complete medium containing hygromycin B (Roche Company, Branford, CT, USA). A gradually increasing dose of hygromycin B (150, 250, 350 and 450 µg/mL) was employed to select the clones with the highest expression of CD20 for further experiments. The overexpression of the CD20 membrane protein was evaluated by flow cytometry (Sysmex Partec GmbH, Görlitz, Sax Germany) using FITC-conjugated anti-CD20 monoclonal antibody (The Dako Group, Glostrup, Denmark) [21].

### 4.2. Aptamer Library and Primers

A ssDNA library was designed, and then synthesized by TAG Copenhagen A/S (Denmark). The synthesized library contained 52 randomized nucleotides (N52) flanked by two 18-nt primer sequences (5′-ATACCAGCTTATTCAATT-N52-AGATAGTAAGTGCAATCT-3′). To amplify the selected aptamer pools, PCR was performed using a forward primer (5′-ATACCAGCTTATTCAATT-3′) and a 5′-phosphate reverse primer (5′-*p*-AGATTGCACTTACTATCT-3′). To prepare high-quality ssDNA from double-stranded DNA, PCR products were digested by lambda exonuclease III (Thermo Fisher Scientific). Another FITC-labeled forward primer (5′-FITC-ATACCAGCTTATTCAATT-3′) was utilized for the amplification of the selected sequences, which were evaluated by flow cytometry.

### 4.3. Cell-SELEX and Aptamer Binding Assay

The ssDNA library was suspended in 100 μL of double-distilled water (DDW). Five nmol from the initial aptamer pool (15 μL, 1016–1018 structure unit) was suspended in 350 μL of the binding buffer. The binding buffer contained 4.5 g of glucose, 100 mg of yeast tRNA (Sigma-Aldrich), 1 g of bovine serum albumin, and 5 mL of 1 M MgCl_2_ dissolved in 1 L of Dulbecco’s PBS (DPBS) (Sigma-Aldrich). Yeast tRNA (0.1 mg/mL) was used to inhibit non-specific binding of aptamers to other target. The reaction mixture (365 μL) was incubated at 95 °C for 5 min and then snap-cooled on ice to enable the formation of secondary structures.

The HEK293T cells (10^6^ cells) that expressed CD20 (CD20 + HEK293T; 90% viable) were suspended in 365 μL of the binding buffer, mixed with the snap-cooled ssDNA library (5 nmol), and incubated on ice for 1 h on a rotary shaker. After centrifugation at 150× *g* for 3 min at 4 °C, the supernatant containing the unbounded sequences was removed. The cell pellet was washed three times with 1 mL of the washing buffer (4.5 g of glucose and 5 mL of 1 M MgCl_2_ in 1 L of DPBS), gently agitated for 30 s, and centrifuged at 150× *g* for 3 min at 4 °C. The bounded ssDNA molecules were eluted from the cell pellet by the addition of 500 μL of DNase-free water in the first round, and the addition of the binding buffer in the subsequent rounds; it was then heated at 95 °C for 5 min. The eluted ssDNA molecules were amplified by PCR using primers specified in above. The PCR products were digested with lambda exonuclease III to obtain an extra-pure ssDNA for the next round of selection. Initially, a preparative PCR was performed to determine the optimum number of PCR cycles that would yield a clear and bright electrophoresis band with no nonspecific amplicons. 

From the second round of cell selection, counter cell selection was also performed, using non-transfected HEK293T cells. This was the negative control. The Cell-SELEX conditions were gradually limited to obtain the most specific aptamers. This was performed by reducing the number of target cells and incubation time, and increasing the wash time and the volume of washing buffer. From the fourth round of selection, FBS concentration was gradually increased from 10% to 20%.

To monitor the specificity of selected aptamers during Cell-SELEX, a flow cytometry binding assay was performed from the fourth round of selection. The experiment was performed using 50 pmol of FITC-labeled selected ssDNA pool in 100 μL of the binding buffer. The mixture was incubated with 106 cells in 200 μL of the binding buffer and 10% FBS, and then placed on ice for 30 min. The cells were then washed two times with the washing buffer. The cell pellet was suspended in 1 mL of the washing buffer, agitated for 30 s, centrifuged at 150 μg for 3 min at 4 °C, and re-suspended in 200 μL of the binding buffer. The fluorescence signal intensity was assessed by flow cytometry analysis of the target and control cells. The unstained cells and cells treated with an unselected FITC library were used to determine the fluorescence background (Auto flourescent).

**Scheme 1:**
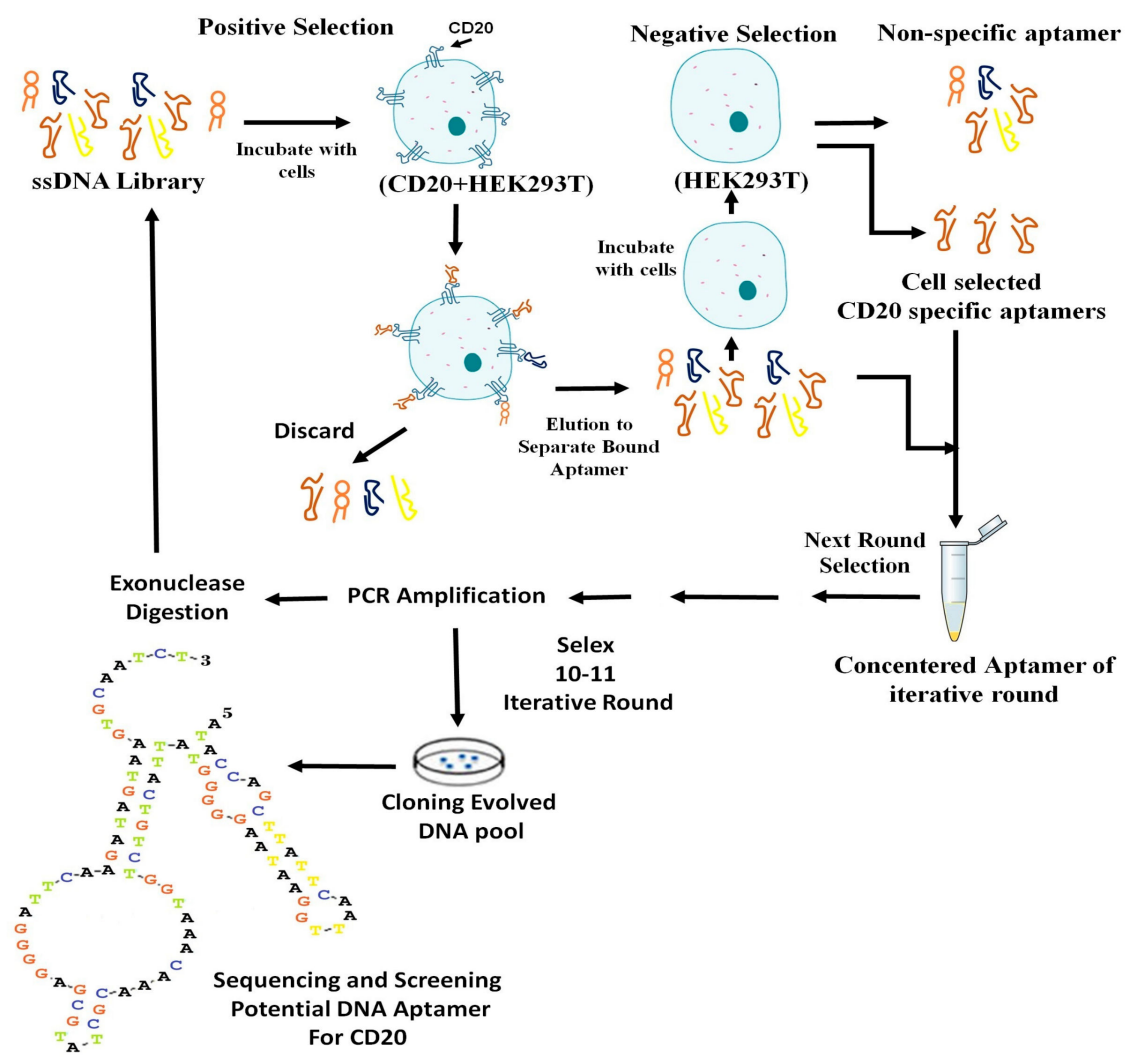
The steps of Cell-SELEX are depicted in the above schematic diagram.

After a sufficient number of rounds of SELEX (10 rounds in the current study), the ssDNA pool from the last round was amplified by PCR. The PCR products were ligated with a T/A cloning vector (Thermo Fisher Scientific) using T4 DNA ligase, and used to transform competent Escherichia coli TOP10 cells (Pasteur Institute, Tehran, Iran). The transformants were selected on Luria Broth (LB) agar plates containing 100 μg/mL ampicillin. The positive clones were evaluated by colony PCR in reactions containing 2 μL of each M13 universal primer (0.5 μM), 0.5 μL of Taq DNA polymerase (5 U/μL), 1 μL of dNTPs (10 mM), 1 μL of MgCl_2_ (100 mM), 5 μL of buffer (10×), DDW (38.5 μL), and the contaminated tip with each colony was washed in PCR tube as a template, in a total reaction volume of 50µL. The PCR was carried out by performing one cycle at 96 °C for 300 s; followed by 34 cycles at 96 °C for 30 s, 42 °C for 30 s, 72 °C for 30 s, and 72 °C for 600 s, in a thermal cycler (Bio-Rad Laboratory, Watford, UK). All of the above steps are schematically depicted in the Scheme 1.

The sixty positive colonies containing plasmids that carried sequences of the selected aptamers were cultured in the LB broth. The plasmids were extracted and sequenced by M13 universal primers (TAG Copenhagen A/S, Frederiksberg, Denmark).

### 4.4. Secondary Structure Estimation

The secondary structures of the aptamers were predicted using the DNAMAN 8.0 software (Lynnon Biosoft, San Ramon, CA, USA) and an aptamer with the highest thermodynamic stability, with the lowest Gibbs free energy (ΔG) (∆G: Kcal/mol), was selected for further assesment.

### 4.5. Calculation of Aptamer Dissociation Constants (K_D_)

Thermodynamically stable aptamers were amplified by FITC primer, and digested using lambda exonuclease III. Serial dilutions of the selected ssDNA aptamers were incubated with HEK293T cells and CD20 + HEK293T cells. The mean fluorescence intensity (MFI) of the aptamer-treated cells with the increasing aptamer concentrations were measured by flow cytometry.

The equilibrium dissociation constant (K_D_) for the aptamer binding affinity of CD20 exposed on the cell membrane was assessed by using the equation Y= B_max_ × X/(K_D_ + X) (X, aptamer concentration; Y, MFI of X; B_max_, maximum MFI). The data were analyzed using the SigmaPlot software (Jandel Scientific, San Rafael, CA, USA), as described previously [28,29]. A comparative analysis of the lowest K_D_ and ΔG of the identified aptamers were performed to select a specific aptamer with the highest binding affinity towards CD20.

### 4.6. Evaluation of Selected Aptamer with Clinical Sample

Two samples of bone marrow from patients with ALL were taken as positive and negative controls. The first sample was selected with Pre-B ALL before treatment. Expression of CD20 was measured by flow cytometry in two ways: once the sample was incubated with anti-CD20 monoclonal antibody labeled with FITC and again was incubated with selected aptamer that was amplified with FITC conjugated primer.

The second sample was selected with T-ALL before treatment. Expression of CD20 was measured by flow cytometry in two ways: once the sample was incubated with anti-CD20 monoclonal antibody labeled with FITC and again was incubated with selected aptamer that was amplified with FITC conjugated primer.

Informed consents for two patients were filled before using their bone marrow (BM) samples.

## 5. Conclusions

The extracellular domain loops of the CD20 protein transfected on HEK293 cells was a suitable target for single-stranded nucleic acid oligomer; aptamers. A library of ssDNA containing 52 randomized nucleotides flanked by two 18-nt primers was constructed to have approximately combination of 10^15^ to 10^18^ aptamers. After 10 rounds of Cell-SELEX the ssDNA pool was amplified by PCR, ligated with pcDNA-3.1/Hygro(+) vector, and used to transform competent *Escherichia coli* TOP10 cells. Finally, three aptamers with the highest thermodynamic stability were selected (AP-1, AP-2 and AP-3). Among them, AP-1 was the most thermodynamically stable aptamer (ΔGAP-1 = −10.87 kcal/mol) with a highest binding affinity (96.91 ± 4.5 nM) to the membrane-expressed CD20 protein. The Cell-SELX aptamers, AP-1, AP2 and AP-3, have similar advantages to antibodies, and could replace instead of antibodies for diagnostic and therapeutic applications. Further studies are required to work towards the use of these aptamers for diagnostic and therapeutic purposes.

## Figures and Tables

**Table 1:** The comparative results of monoclonal anti-CD20 antiboby and AP-1 aptamer for two patients with Pre-B ALL and T-ALL with and without expressing CD20 as positive and negative control, respectively.

Test	Anti-CD20 Antibody	AP-1 Aptamer
CD 20 positive(A bone marrow of patient with Pre-B ALL before treatment)	84.4%	71.4%
CD 20 negative(A bone marrow of patient with T-ALL before treatment)	6.5%	1.5%
